# Ultrafast transient liquid assisted growth of high current density superconducting films

**DOI:** 10.1038/s41467-019-13791-1

**Published:** 2020-01-17

**Authors:** L. Soler, J. Jareño, J. Banchewski, S. Rasi, N. Chamorro, R. Guzman, R. Yáñez, C. Mocuta, S. Ricart, J. Farjas, P. Roura-Grabulosa, X. Obradors, T. Puig

**Affiliations:** 1 Institut de Ciència de Materials de Barcelona, ICMAB-CSIC, Campus UAB, 08193 Bellaterra, Catalonia Spain; 2 GRMT, Department of Physics, Universitat de Girona, Campus Montilivi, Edif. PII, E17003 Girona, Catalonia Spain; 3 Departament de Química, Universitat Autònoma de Barcelona, 08193 Bellaterra, Catalonia Spain; 4 Synchrotron SOLEIL, L’Orme des Merisiers Saint-Aubin BP 48, 91192 Gif-sur-Yvette, France

**Keywords:** Superconductors, Electronic properties and materials, Superconducting properties and materials

## Abstract

The achievement of high growth rates in YBa_2_Cu_3_O_7_ epitaxial high-temperature superconducting films has become strategic to enable high-throughput manufacturing of long length coated conductors for energy and large magnet applications. We report on a transient liquid assisted growth process capable of achieving ultrafast growth rates (100 nm s^−1^) and high critical current densities (5 MA cm^−2^ at 77 K). This is based on the kinetic preference of Ba-Cu-O to form transient liquids prior to crystalline thermodynamic equilibrium phases, and as such is a non-equilibrium approach. The transient liquid-assisted growth process is combined with chemical solution deposition, proposing a scalable method for superconducting tapes manufacturing. Additionally, using colloidal solutions, the growth process is extended towards fabrication of nanocomposite films for enhanced superconducting properties at high magnetic fields. Fast acquisition in situ synchrotron X-ray diffraction and high resolution scanning transmission electron microscopy (STEM) become crucial measurements in disentangling key aspects of the growth process.

## Abstract (web summary)

High throughput manufacturing of long length coated conductors requires fast epitaxial growth of high-temperature superconducting films. Here, Soler et al. report an ultrafast growth rates and high critical current densities of YBa_2_Cu_3_O_7_ films using a transient liquid-assisted growth method.

## Introduction

The discovery of cuprate superconductors, 30 years ago, raised enormous hope for widespread use of superconductivity in many large-scale energy applications and powerful magnets. However, huge challenges needed to be addressed to provide a flexible conductor with high current transport capabilities. Coated conductors (CCs) revolutionized this area by growing epitaxial REBa_2_Cu_3_O_7_ (REBCO, RE = Rare Earth or Y) layers on top of buffered long length flexible metallic substrates. At present, they are the materials with highest current capabilities and the most promising to extend large-scale applications of superconductivity beyond those achieved with low-temperature superconductors^1–3^. Remarkable relevance was achieved by the growth of nanocomposites^4–7^, which strongly enhanced performances at high magnetic fields. However, the low throughput in the production of CCs remains a severe limitation to generate drastic cost reductions of CCs for wide market implementation.

Chemical solution deposition (CSD) is a non-vacuum approach leading to CCs with very competitive performance, while having a strong potential for achieving the above-mentioned cost reductions. Nevertheless, the low throughput owing to small growth rates (∼1 nm s^−1^) from the most extended trifluoracetate-route (TFA)^8^ still restricts from full development of CSD potentialities.

The approach identified to reach high film growth rates, is that based on liquid-assisted growth^9–11^. In this case, fast atomic diffusion and high atomic density encompass a much higher growth rate than in vapor or solid diffusion reaction approaches^12^. More recently, few examples of liquid-assisted growth of REBCO films are found, all based on vacuum deposition techniques^10,11,13–16^.

Here, we report on an outstanding process that goes beyond the established schemes based on growth from liquids appearing in the equilibrium phase diagram. It combines CSD using non-fluorinated precursors and a non-equilibrium process grounded on transient liquids, originating an ultrafast growth approach: Transient Liquid-Assisted Growth based on CSD (TLAG-CSD). The kinetic hindrances in crystallization are advantageously used to induce transient liquids, i.e., liquids that do not exist in the equilibrium ternary phase diagram for such compositions. This idea results in growth rates of ∼ 100 nm s^−1^ for epitaxial YBCO films, as demonstrated in this work by in situ synchrotron X-ray diffraction studies. In addition, the *T–P*_O2_ window for heteroepitaxial growth is strongly enlarged and the process is shown to be compatible with scalable solution deposition techniques. We further prove that TLAG-CSD nanocomposite films, with high critical temperatures and current densities, can be grown with the above depicted approach using colloidal solutions of 5 nm-size BaMO_3_ (M = Zr, Hf) preformed nanoparticles. These nanocomposites display relevant nanostructural changes that positively influence vortex pinning at high magnetic fields. The synergistic effect between nanoparticles and local nanoscale lattice distortions (nanostrain) observed, brings additional opportunities to further improved vortex pinning efficiency of TLAG-CSD nanocomposites. In addition, TLAG-CSD brings opportunities for simplified reactors and precursors flexibility, thus it is compliant with a high-throughput method for easier market penetration.

## Results

### TLAG principles

**Fig. 1:**
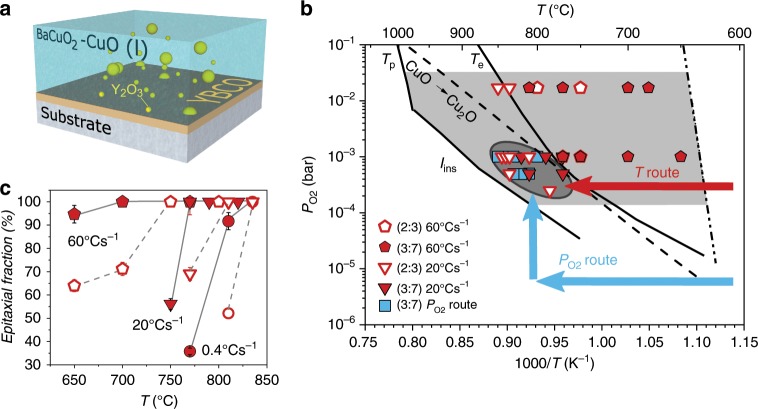
Transient liquid-assisted growth (TLAG) principles. **a** Schematic drawing of the TLAG process. **b** *T–P*_O2_ phase diagram for YBCO TLAG growth. The 2BaCuO_2_-CuO eutectic temperature, *T*_e_, the YBCO peritectic temperature, *T*_p_, and the instability YBCO line, *I*_ins_, are shown. The T-route and P_O2_-route are indicated by arrows. Symbols are the final temperature and *P*_O2_ values of individual experiments: open symbols (2BaCuO_2_-CuO, 2:3 composition) or closed symbols (3BaCuO_2_-4CuO, 3:7 composition) in red (T-route) or blue (P_O2_-route); all symbols indicate 100% epitaxial growth. *T*_e_ has been determined from ref. ^27^ and this article (see details in methods); *T*_p_, *T*_ins_, and the CuO-Cu_2_O line are from ref. ^10^. **c** Epitaxial fraction dependence on growth temperature for the T-route at different heating rates for 2:3 (empty symbols) and 3:7 (solid symbols) compositions at *P*_O2_ = 10^−3^ bar. Error bars indicate the accuracy of the method as determined in ref. ^7^.

The TLAG process is based on the capability of the ternary system BaCuO_2_, CuO, and Y_2_O_3_ to achieve ultrafast growth of YBCO films. This is possible because the eutectic reaction between the first two compounds is able to form a transient liquid (with the presence of dispersed Y_2_O_3_ solid nanoparticles) in the region of the phase diagram where solid YBa_2_Cu_3_O_7-x_ is the equilibrium phase (light gray region of Fig. 1b). The idea is that there is an energy barrier for nucleation of the crystalline phase (which delays its formation through an incubation time), whereas the transient liquid phase can emerge instantaneously, thus enabling the development of an ultrafast non-equilibrium processing approach^17^.

Upon dissolution of the Y_2_O_3_ nanoparticles in the Ba-Cu-O transient liquid and Y diffusion toward the substrate interface (see diagram of Fig. 1a), epitaxial growth of YBCO occurs. The fast Y diffusion in the liquid may boost the growth rate. For instance, the diffusivity of Y at 970 °C in the BaCuO_2_-CuO liquid is 4 × 10^−10^ m^2^ s^−1^^18^, whereas in the YBCO solid is almost two decades lower, of only 8 × 10^−12^ m^2^ s^−1^^19^. This makes liquid-assisted processes potentially so much faster than solid–solid or solid–gas reactions. We anticipate that the crystallization through the liquid will be mostly limited by the substrate interfacial kinetics of Y, Ba, Cu, and O atoms^20,21^.

Our results demonstrate that crystallization rates of at least 100 nm s^−1^ are reachable. In addition, we show that the liquid can be formed below the binary BaCuO_2_-CuO equilibrium eutectic temperature, *T*_e_ (Fig. 1b), thus TLAG-YBCO epitaxial growth is achieved in a wide region of the phase diagram (light gray region of Fig. 1b). In summary, the kinetic crystallization hindrances are exploited to induce much faster crystallization through a transient liquid.

In general, epitaxial film growth is ensured by properly tuning the supersaturation conditions. In the TLAG case, liquid composition^11,22^ and RE element solubility in the melt^23^ are the most relevant parameters. Several process parameters (temperature, oxygen partial pressure *P*_*O2*_, total pressure of gases in the reactor *P*_total_, heating ramp rates) have to be optimized for epitaxial growth for each composition. In this work, two different liquid compositions were studied, 2BaCuO_2_-CuO (2:3) and 3BaCuO_2_-4CuO (3:7), which correspond to the YBa_2_Cu_3_O_x_ and YBa_2_Cu_4.66_O_x_ film compositions, respectively. The latter matches the Ba-Cu-O eutectic liquid composition and results in the desired phase (YBa_2_Cu_3_O_x_) together with CuO nanoparticles, mainly observed at the film surface. Both melts give rise to epitaxial YBCO films.

Our results reveal that the temperature window for *c* axis epitaxy is widened to temperatures below *T*_e_ and that this expansion depends on the heating ramp (down to 650 °C for the 3:7 composition at *dT/dt* = 60 °C/s, Fig. 1b, c). In fact, as TLAG is a non-equilibrium kinetically driven process, when high heating rates are used, the Y ions dissolution from the Y_2_O_3_ nanoparticles into the melt might be compromised, and the Y concentration in the liquid, *C*_Y_, will decrease. As a consequence, the relative supersaturation of the liquid, *σ* = (*C*_Y_/*C*_e_−1) (where *C*_e_ is the equilibrium concentration), will decrease, leading to the enhanced *c* axis nucleation and growth window observed. This fact brings unique opportunities for fine tuning of the film manufacturing process by employing rapid thermal annealing furnaces.

When the two liquids are compared, one realizes that the 3:7 eutectic composition is able to trigger the formation of *c* axis YBCO at lower temperatures (solid versus open symbols of Fig. 1b, c). This behavior is in agreement with a lower supersaturation for the 3:7 liquid composition. If *C*_Y_ is assumed similar for both liquids, since *C*_e_ increases with Cu content in the liquid^24^, supersaturation will indeed be lower for the 3:7 liquid composition^25^.

**Fig. 2:**
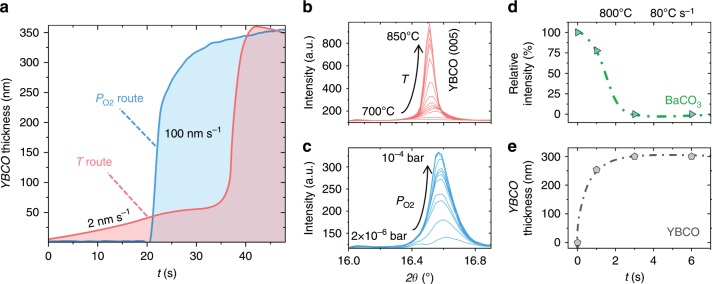
Fast evolution of phases in TLAG by in situ XRD synchrotron radiation. **a** YBCO growth rate determined from in situ XRD synchrotron analysis of a 350 nm film demonstrating 100 nm s^−1^ growth rate for T-route (*P*_O2_ = 10^−3^ bar, *dT/dt* = 5 °C s^−1^) and *P*_O2_-route (*T* = 850 °C, *P*_O2,base_ = 10^−5^ bar, *P*_O2,final_ = 10^−2^ bar). Initial stages for the T-route show a growth rate of 2 nm s^−1^. **b**, **c** Evolution of the diffracted intensity of the (005) YBCO peak for the T-route and P_O2_-route, respectively, during the transformation for the experiment reported in **a**. The X-ray energy used for these measurements was18 keV. The evolution with time of the XRD intensity for **d** BaCO_3_ and **e** YBCO phases in a T-route process in a RTA furnace at 800 °C (*P*_O2_ = 10^−2^ bar, *dT/dt* = 80 °C s^−1^) for a film of 300 nm. Notice that BaCO_3_ consumption to form the transient liquid goes in parallel with YBCO growth, which reaches completion in 3 s in these conditions.

The best conditions achieved so far, demonstrating high critical current density in the superconducting films, are shown in Fig. 1b as the dark gray encircled region (for both compositions and different TLAG routes investigated in this work, see details below). It is worth noting that reaching this high-temperature region in a very short time lapse is very important because here is where the growth rate is larger: ultrahigh values in the range of 100 nm s^−1^ have been demonstrated for *T* > *T*_e_, whereas two orders of magnitude lower growth rates (2 nm s^−1^) are reached at lower temperatures (see Fig. 2a). This is further confirmed in Fig. 2e, where the fast growth of TLAG is obtained from rapid thermal annealed samples quenched at 800 °C, demonstrating that 3 s are enough to grow a 300 nm YBCO film (i.e., 100 nm s^−1^) with a heating ramp of 80 °C s^−1^.

### TLAG-CSD approach

The potential for high-throughput and low-cost TLAG-CSD CCs is based on the idea of combining the ultrafast growth of TLAG with CSD. A stable and atomically homogeneous solution of Y, Ba and Cu salts based on propionate precursors is deposited on a substrate and pyrolyzed to reach a homogeneous nanoporous layer of Y_2_O_3_, BaCO_3_ and CuO nanocrystalline phases (details given in Methods). Then, the sample is heated up to the gray region of Fig. 1b, where upon BaCO_3_ reaction, a homogeneous dispersion of Y_2_O_3_(s) nanoparticles is formed in the BaCuO_2_-CuO (liquid), to crystallize into epitaxial YBCO.

The TLAG-CSD process is compatible with multideposition of pyrolyzed layers and scalable ink jet printing patterning techniques, where layer thickness is not only controlled by the solution concentration but also by drop volume and drop spacing^26^. Pyrolyzed layers up to 1 µm thick deposited by ink jet printing show a high degree of homogeneity throughout thickness (see Supplementary Figure 1a). These layers are transformed after the TLAG growth process to homogeneous, pore free, and epitaxial YBCO films of 550 nm (Supplementary Figure 1b), where straight twin boundaries crossing the total film thickness can be identified. Higher magnification with atomic resolution scanning transmission electron microscopy (STEM) confirms the highly crystalline quality of the epitaxial growth.

The versatility and speed of the TLAG-CSD growth process has been further investigated by in situ fast acquisition x-ray diffraction analysis at SOLEIL synchrotron (Fig. 2 and Supplementary Figure 2) and advanced electron transmission microscopy from quenched samples (Supplementary Figure 3). Both techniques enabled us to disentangle the phase transformations and growth mechanisms. A key point to understand the TLAG-CSD growth mechanism was the identification of the reaction of BaCO_3_ with Cu-phases to form the Ba-Cu-O intermediate phases.

We have investigated two different routes indicated in Fig. 1b with arrows, temperature route (T-route, with red arrows) and oxygen partial pressure route (P_O2_-route, with blue arrow). In the T-route, BaCO_3_ reacts with CuO during heating at a fixed oxygen partial pressure (10^−4^ bar < *P*_O2_ < 10^−2^ bar); then the transient liquid forms dissolving Y ions from Y_2_O_3_ to give rise to YBCO (see in situ X-ray powder diffraction (XRD) synchrotron studies reported in Supplementary Figure 2). This reaction process is represented in Eq. (1) (not adjusted since we have used two considerably different liquid compositions: 2BaCuO_2_-CuO (2:3) and the 3BaCuO_2_-4CuO (3:7)).

1 $${\mathrm{BaCO}}_{\mathrm{3}}\left( {\mathrm{s}} \right)	{\mathrm{ + CuO}}\left( {\mathrm{s}} \right){\mathrm{ + Y}}_{\mathrm{2}}{\mathrm{O}}_{\mathrm{3}}\left( {\mathrm{s}} \right) \to \left[ {{\mathrm{Ba - Cu - O}}} \right]\left( {\mathrm{l}} \right){\mathrm{ + Y}}_{\mathrm{2}}{\mathrm{O}}_{\mathrm{3}}\left( {\mathrm{s}} \right) \\ 	\to {\mathrm{YBa}}_{\mathrm{2}}{\mathrm{Cu}}_{\mathrm{3}}{\mathrm{O}}_{{\mathrm{6}}{\mathrm{.5}}}\left( {\mathrm{s}} \right){\mathrm{ + xCuO}}\left( {\mathrm{s}} \right)$$

where the labels (*s*), (*l*) stand for solid and liquid, respectively, and *x* = 0 is for (2:3) and *x* ≠ 0 for (3:7) compositions used.

In the P_O2_-route (blue arrows of Fig. 1b), BaCO_3_ reacts during heating at low (and constant) oxygen partial pressure (10^−5^ bar *<* *P*_O2,base_ < 10^−4^ bar) in the region where YBCO is not the stable phase (i.e., below the instability line, *I*_ins_, of Fig. 1b), following the reaction process shown in Eq. (2). Then, a steep increase in *P*_O2_ (10^−4^ bar < *P*_O2,final_ < 10^−2^ bar) at constant temperature, brings the film to the right region of *T–P*_O2_ in order to form the transient Ba-Cu-O liquid that allows fast epitaxial growth of YBCO (Eq. (3)). Hence, in this route, the elimination of BaCO_3_ is fully decoupled from the YBCO growth.

According to Eq. (2), initial BaCO_3_ and Cu_2_O react to generate BaCu_2_O_2_, which is the stable solid Ba-Cu phase at this low *P*_O2_^27,28^, (Supplementary Figure 2c).

2 $${\mathrm{BaCO}}_{\mathrm{3}}\left( {\mathrm{s}} \right)	{\mathrm{ + CuO}}\left( {\mathrm{s}} \right){\mathrm{ + Y}}_{\mathrm{2}}{\mathrm{O}}_{\mathrm{3}}\left( {\mathrm{s}} \right) \to {\mathrm{BaCO}}_{\mathrm{3}}\left( {\mathrm{s}} \right){\mathrm{ + Cu}}_{\mathrm{2}}{\mathrm{O}}\left( {\mathrm{s}} \right){\mathrm{ + Y}}_{\mathrm{2}}{\mathrm{O}}_{\mathrm{3}}\left( {\mathrm{s}} \right) \\ 	\to {\mathrm{BaCu}}_{\mathrm{2}}{\mathrm{O}}_{\mathrm{2}}\left( {\mathrm{s}} \right){\mathrm{ + Y}}_{\mathrm{2}}{\mathrm{O}}_{\mathrm{3}}\left( {\mathrm{s}} \right)$$

After the steep increase in oxygen partial pressure (*P*_O2_ *=* *P*_O2,final_) at constant temperature, the BaCu_2_O_2_ solid phase transform to Ba-Cu-O liquid and subsequent YBCO growth (Supplementary Figure 2d), following the reaction process given in Eq. (3). Again Eq. (2, 3) are not adjusted to generalize in the liquid compositions employed. Notice that, for the mechanism detailed in Eq. (2, 3), *P*_*O2*_ is the crucial parameter, whereas *P*_total_ was kept between 10^−5^ and 10^−2^ bar.

3 $${\mathrm{BaCu}}_{\mathrm{2}}{\mathrm{O}}_{\mathrm{2}}\left( {\mathrm{s}} \right)	{\mathrm{ + Y}}_{\mathrm{2}}{\mathrm{O}}_{\mathrm{3}}\left( {\mathrm{s}} \right) \to \left[ {{\mathrm{Ba - Cu - O}}} \right]\left( {\mathrm{l}} \right){\mathrm{ + Y}}_{\mathrm{2}}{\mathrm{O}}_{\mathrm{3}}\left( {\mathrm{s}} \right) \\ 	\to {\mathrm{YBa}}_{\mathrm{2}}{\mathrm{Cu}}_{\mathrm{3}}{\mathrm{O}}_{{\mathrm{6}}{\mathrm{.5}}}\left( {\mathrm{s}} \right){\mathrm{ + xCuO}}\left( {\mathrm{s}} \right)$$

where *x* = 0 stands for (2:3) and *x* ≠ 0 for (3:7) liquid composition.

In this P_O2_-route case, ultrafast growth rates of 100 nm s^−1^ have also been reached (Fig. 2a) by controlling the steepness of the *P*_O2_ change. Direct proof of the transient liquid phase for both, T- and P_O2_-routes, is given by the results of in situ XRD experiment reported in Supplementary Figure 2) where the crystalline precursor phases disappear at the onset of YBCO growth.

A detailed TEM analysis of the TLAG process for the T-route approach is shown in Supplementary Figure 3, where the stages of the process with the different reaction phases and their distribution are presented. The initial nanocrystalline phases observed after the pyrolysis (BaCO_3_, CuO, and Y_2_O_3_) are of tens of nanometers in size, so fast reaction paths are rendered possible. The CuO nanoparticles, of size in the range of 10–30 nm, are distributed within a BaCO_3_ matrix. At an intermediate temperature step, the first heterogeneous nuclei of YBCO grow at the substrate interface whereas BaCO_3_ grains (size ~ 40-50 nm) remain in the bulk of the film. This confirms that the slowest reaction is the BaCO_3_ elimination. At the final stage, the YBCO epitaxial layer is observed and BaCO_3_ has completely disappeared. The BaCO_3_ reaction time is a key point in this process and strongly depends on heating rate and final temperature. At 80 °C s^−1^ and 800 °C, for instance, only 3 s are needed to completely remove BaCO_3_ and to grow a 300 nm thick YBCO film (Fig. 2d, e). In some particular cases, homogeneously nucleated YBCO nanocrystals can eventually recrystallize, as suggested by synchrotron XRD analysis (Supplementary Figure 2a).

We conclude that TLAG-CSD is an ultrafast non-equilibrium and versatile process to grow epitaxial YBCO films at *c* axis growth rates of ∼ 100 nm s^−1^ from different routes. This is ~10–100 times faster than most of the thin film processes presently used^8,29–32^ to grow coated conductors and it confirms fast growth rates observed in other liquid-assisted techniques, such as in REBCO single crystal growth, REBCO bulk ceramic melt-textured growth^33^ or REBCO films^14^. However here, the process is not restricted to the use of an equilibrium liquid phase and we demonstrate that the transient liquid formation is compatible with high-throughput CSD film manufacturing.

### TLAG-CSD film properties

**Fig. 3:**
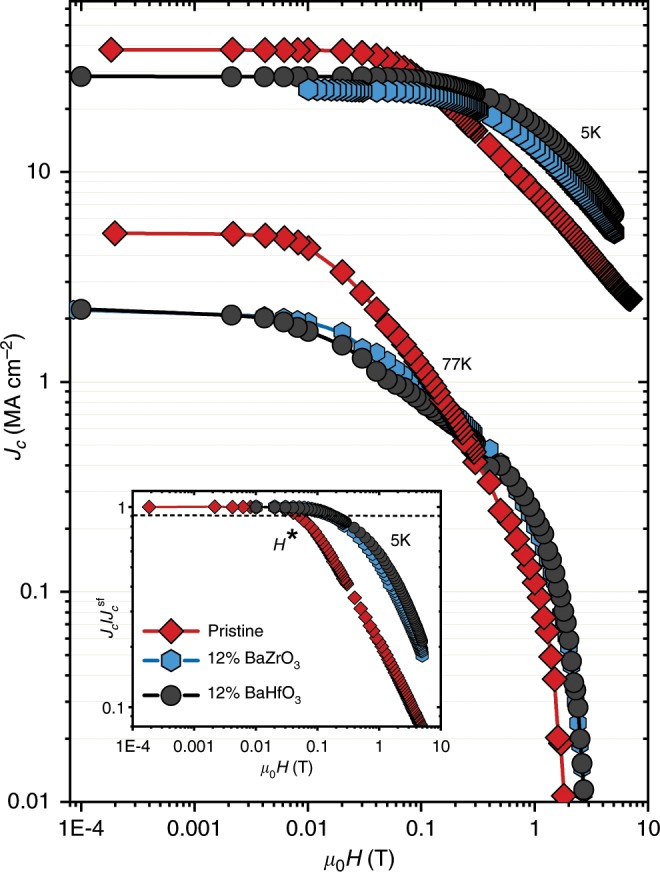
Inductive critical current density, *J*_c_, as a function of the magnetic field for TLAG films. A pristine YBCO film and two nanocomposites of 12% molar BZO and 12% molar BHO of composition 3:7 are shown for 5 K and 77 K. The growth conditions followed the P_O2_-route at temperature of 835 °C and *P*_O2,final_ = 10^−3^ bar. Samples are 90 nm thick. The inset shows the normalized *J*_c_ for the self-field value at 5 K for the same three films. The H* parameter is defined with the criterion of the value of H at 0.9 times *J*_c_ at self-field. The irreversibility line obtained from electrical transport measurements is shown in Supplementary Figure 6, displaying an µ_0_H_irr_(77 K) ~ 8.1 T.

Optimized conditions for the TLAG-CSD process gives rise to highly epitaxial YBCO films of 200–300 nm thickness (out-of-plane and in-plane textures of Δω < 0.3° and Δφ < 0.5°, respectively, Supplementary Figure 4). Also, very high critical current densities of 5 MA cm^−2^ at 77 K and 39 MA cm^−2^ at 5 K with transition temperature ~ 90 K are reached (Fig. 3 and Supplementary Figure 5) upon tuning the process parameters (*P*_total_, *P*_O2_, *T*, ramp rates, substrate). The particular case shown in Fig. 3 is for a 90 nm thick YBCO film using the P_O2_-route with the reaction mechanism of Eq. (2, 3).

Liquid-assisted growth, however, exposes some challenges that need to be properly addressed for each type of route. Coarsening of unreacted precursor phases in the liquid, liquid reactivity with the substrates (or the buffer layers) owing to its highly corrosive nature and, improper substrates wettability with the viscous liquid^34^ or trapped liquid at grain boundaries, are some of them. Nevertheless, we have proved that superconducting films with very high critical current densities can be grown if the aforementioned obstacles are overcome, in agreement with previous liquid-assisted CCs growth^14,25^.

**Fig. 4:**
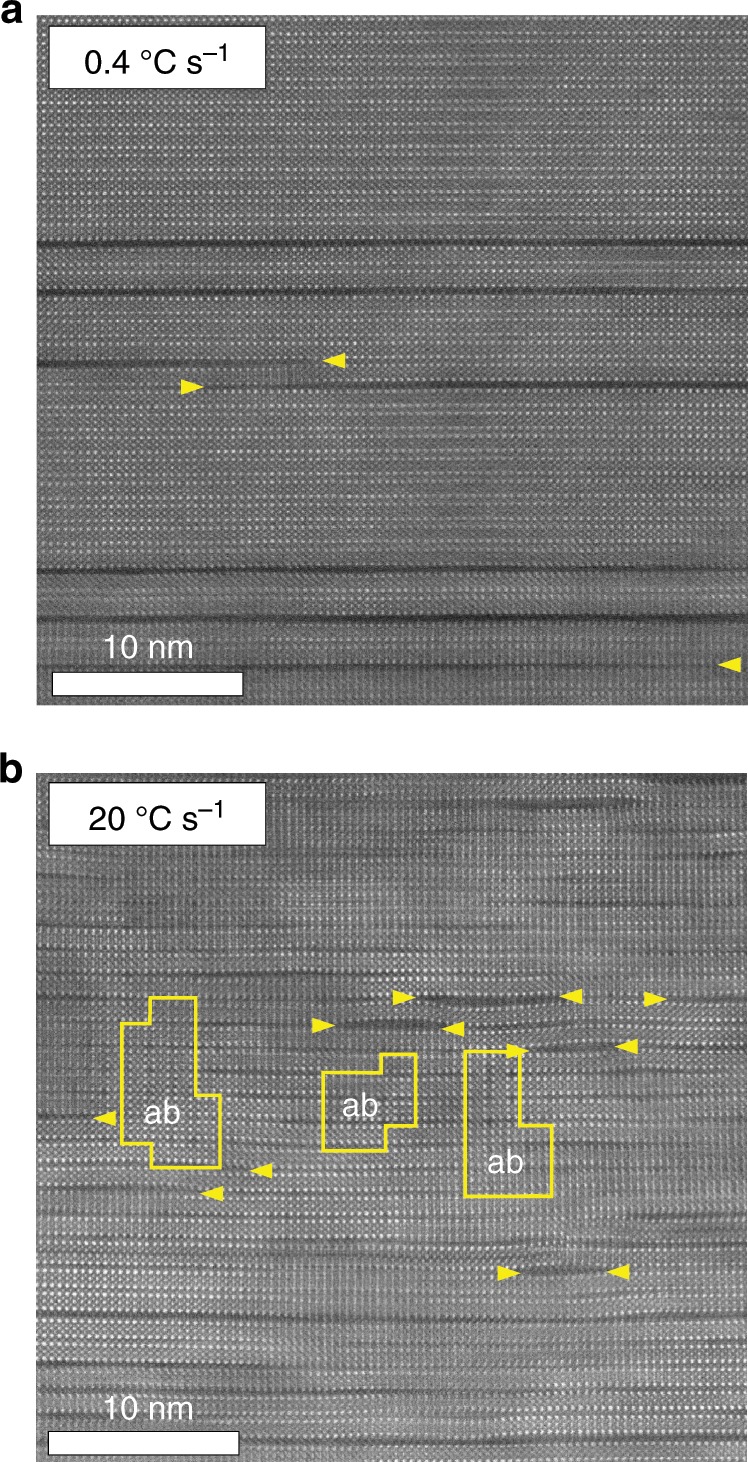
STEM cross-section images of TLAG-YBCO films highlighting the differences in microstructure for the T-route. **a** at a heating rate of 0.4 °C s^−1^, where highly epitaxial film with a low density of defects (Y248 intergrowth) is shown, and **b** at 20 °C s^−1^ heating rate, that shows a highly distorted YBCO matrix with a high density of short Y248 intergrowths and nanoscale ab grains. Yellow arrows indicate some of the Y248 intergrowths.

Figure 4 shows a cross-section STEM image demonstrating the quality of the film growth. Each route has a distinctive microstructure. However, the main defects are the Y_2_Ba_4_Cu_8_O_x_ (Y248) intergrowths (double CuO chains), similarly to the TFA-CSD process^35^. For the T-route, at low heating rates of 0.4 °C s^−1^ a microstructure with only few Y248 intergrowth (indicated by yellow arrows) is observed (Fig. 4a), in agreement with the low growth rate identified at low temperatures, where most of the film would grow. At increased heating rates of 20 °C s^−1^, we observe a high density of defects, mainly double and triple short CuO chain intergrowths (also marked with yellow arrows) and nanoscale ab grains in the range of 5–10 nm distributed in the bulk of the film (Fig. 4b). This is in agreement with the ultrafast TLAG-CSD growth process achieved at high temperatures. Overall, we want to highlight that different microstructures are reached depending on the process route and parameters, making TLAG-CSD a very tuneable process for vortex pinning.

### Growth of TLAG nanocomposites

**Fig. 5:**
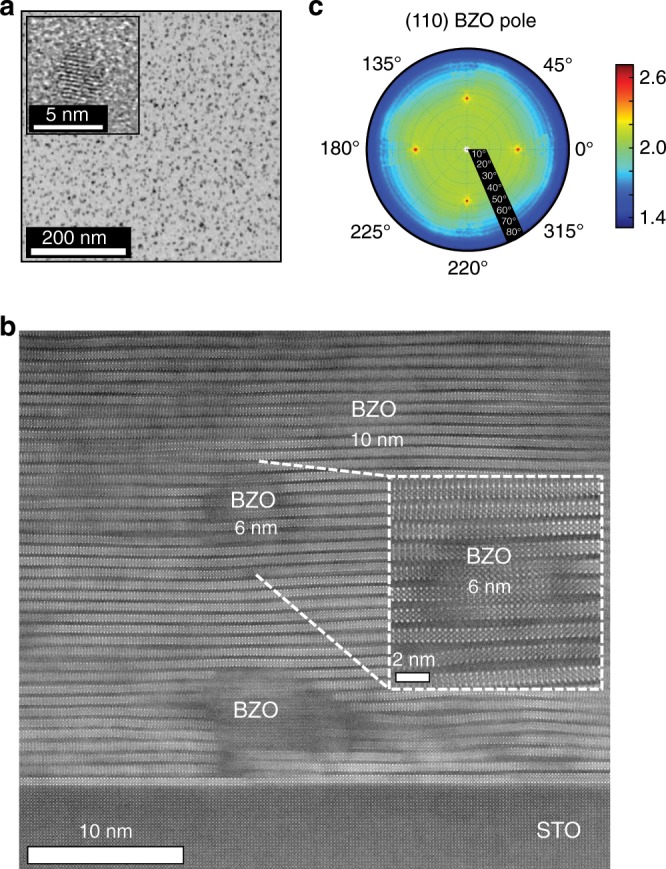
Growth of TLAG nanocomposites. **a** TEM image of 5 nm BaHfO_3_ nanoparticles in the colloidal solution. **b** STEM image of a 12% Molar BaZrO_3_ (BZO) nanocomposite (in a 90 nm thick YBCO layer) on SrTiO_3_ single crystal substrate, where few BZO nanoparticles are identified. The inset shows a high resolution image of the region with a 6 nm BZO nanoparticle embedded in the YBCO matrix; strong lattice distortions and Y248 intergrowth generated are evidenced. **c** (110) Pole figure of the 24% molar BZO nanocomposite where epitaxial orientation of the nanoparticles is demonstrated (data are fourfold symmetrized, XRD analysis performed at SOLEIL Synchrotron).

The growth of nanocomposites has demonstrated to be the path to pin vortices and reach high critical currents at high magnetic fields in coated conductors, especially for large magnet applications^4–7,36,37^. In CSD, most of the effort has been done on adding metalorganic salts to the precursor solution; a method that relies on the spontaneous segregation process of nanoparticles during YBCO growth^37,38^. However, this approach is rather complex with TLAG-CSD, as one would need to finely control two ultrafast quasi-simultaneous nucleation processes. Instead, we demonstrate that TLAG-CSD is compatible with nanocomposites growth when preformed nanoparticles are stabilized in the precursor solution. This result represents a second breakthrough for the TLAG-CSD process, beyond the ultrafast growth. In this case, a colloidal solution of BaMO_3_ (BMO, *M* = Zr, Hf) nanoparticles of 5 nm in diameter (Fig. 5a) was stabilized in the Y, Ba, Cu propionates-based metalorganic solution, in a similar way as for TFA-CSD^39^. Cross-section STEM images of a nanocomposite film, grown following the P_O2_-route, and its associated microstructure are shown in Fig. 5b. Notice that the final nanoparticles size, identified by STEM measurements, is in the range of 6–10 nm. These values have also been confirmed by XRD crystallite size analysis, evidencing a low degree of coarsening. Nanocomposites with large nanoparticle content (up to 32% mol) have been prepared maintaining high *T*_c_ in the range of 88–92 K and epitaxial growth (Supplementary Figure 4 and 5). The self-field critical current density starts to decrease for nanocomposites with nanoparticles contents beyond 24% mol, which can be explained by some agglomeration of the nanoparticles and minor non-oriented grains.

A remarkable feature of TLAG-CSD nanocomposites is that BMO nanoparticles tend to grow epitaxially with the YBCO matrix (see (110) pole-figure of BZO in Fig. 5c). This effect is similar to that observed in techniques based on simultaneous deposition and growth (PLD, MOCVD)^36,40^ and it is opposite to the TFA-CSD route where nanoparticles are found randomly oriented^7,37^. Quantification of the epitaxial fraction following the method reported in^7^ gives an epitaxial fraction in the range from 85 to 95% for the TLAG-CSD nanocomposites. This demonstrates that the liquid phase allows nanoparticles rotation inside the liquid to minimize interfacial energies. This rotation actually occurs simultaneously to an enhanced crystallinity and some recrystallization of the BMO nanoparticles (Fig. 5b). On the contrary, in the TFA-CSD process, the solid-state hinders the rotation of the nanoparticles, i.e., nanoparticles remain randomly oriented, even if some recrystallization leading to faceted interfaces was also observed^7^.

We also point out that the density of Y248 intergrowths is extremely high in TLAG nanocomposites (Fig. 5b). This indicates that the driving force for Y248 formation in this process is not provided by the minimization of large incoherent interfaces like in the TFA-CSD case^7^. Instead, we propose that the highly defective YBCO matrix is induced by the ultrafast growth of the nanocomposite film. It is known that the Y248 intergrowth is one of the lowest energy defects able to accommodate large distortions in YBCO^35^. The high density of local nanoscale distortions can be characterized by XRD Williamson–Hall analysis in terms of nanostrain^7^. The corresponding increase of the nanostrain with the density of nanoparticles (Supplementary Figure 5) is in agreement with previous analysis on TFA-CSD nanocomposites, where local strained regions serve as strong artificial pinning centers due to strain induced Cooper-pair suppression^7^.

Figure 3 illustrates the effect of nanoparticles and associated microstructure on the magnetic field dependence of the critical current density, *J*_c_, for a 12% mol of BZO and 12% mol BHO nanocomposite films of 90 nm thickness. Notice that the critical current density of TLAG-CSD nanocomposites out-performs that of pristine samples already above a magnetic field of 0.1–0.2 T, even though the self-field values were not properly optimized yet. The much smoother in-field *J*_c_ dependence remains for the whole-temperature range and the crossover field identified by the H* parameter (see Fig. 4 inset) can even reach values of 200 mT, which are on the order of the highest results measured by SQUID in CSD YBCO nanocomposite films^39^. For the same values of nanostrain and therefore of local distortion, nanocomposites present a large increase of H* with respect to pristine samples (Supplementary Figure 5), showing that there is another pinning mechanism acting beyond the nanostrain effect. We suggest that nanoparticles of 6–8 nm (diameter 2–3 times higher than the vortex coherence length, *ξ*_ab_) could be directly pinning vortices^41^, thus the aforementioned H* enhancement in TLAG nanocomposites might be partially governed by nanoparticles acting themselves as core pinning centers. Such a scenario might pave the way to radical performance increase in CSD nanocomposite films upon continuing nanoparticle miniaturization and the prevention of coarsening at high concentrations. We emphasize that the fast crystallization kinetics of the TLAG approach can advantageously be used to push the frontier of nanocomposite-coated conductor manufacturing.

## Discussion

We have developed a non-equilibrium process to grow YBCO films and nanocomposites at ultrafast growth rates (∼ 100 nm s^−1^), which can be combined with low-cost CSD and large area ink jet printing deposition techniques. The breakthrough idea is the use of a transient liquid to grow epitaxial YBCO films. Critical current densities of 5 MA cm^−2^ at 77 K and in-field enhanced properties for nanocomposites based on colloidal nanoparticles solutions have been demonstrated. This growth technique appears very promising for high-throughput preparation of YBCO films and if demonstrated for long length thick coated conductors, it could widely spread the use of high-temperature superconducting materials for practical applications. Preliminary work on growth of 1 µm thick TLAG-CSD films show no difficulties in eliminating BaCO_3_ in any of the routes here reported, as well as no reactivity on LaMnO_3_-buffered coated conductors. In our opinion, this suggests a great future for this methodology, easily scale-up and integrated in simple reel-to-reel furnaces, to create a high-throughput and low-cost CC manufacturing schemes for fabrication of coated conductors. It is based on very general kinetic and thermodynamic principles and could therefore be applied to grow other functional nanomaterials.

## Methods

### Sample preparation

Epitaxial *c* axis-oriented YBCO and BMO (*M* = Zr, Hf) nanocomposites thin films have been grown on single-crystalline (100) SrTiO_3_ substrates. Samples were prepared from metalorganic precursor solutions with two stoichiometries: YBa_2_Cu_3_O_x_ and YBa_2_Cu_4.66_O_x_. Y, Ba, and Cu anhydrous acetates were dissolved in propionic acid for four hours at 50 °C and afterwards anhydrous methanol (in a 50/50) mixer was added to reach a 1 M–1.5 M concentration depending on desired film thickness. In some cases, a 5%_v/v_ triethanolamine was also added to reach larger thickness. Films of thickness from 90 nm up to 500 nm were studied. Spin coating was used for films up to 300 nm thickness with a pyrolysis of 500 °C for 5 min in an oxygen atmosphere, whereas a home-made ink jet printer with microfab piezoelectric nozzles was used for film thickness from 300 up to 500 nm. Superconducting YBCO films were grown in a Rapid Thermal Annealing furnace from Annealsys AS.Micro equipped with a SiC coated graphite susceptor, capable of reaching heating rates up to 80 °C s^−1^ with calibrated temperatures. The T-route or P_O2_-route explained in the main text were used as convenient. In the T-route, temperature was increased up to a range between 650 and 850 °C at a heating rate between 0.4 °C s^−1^ and 80 °C s^−1^, at constant oxygen partial pressure between 5 × 10^−4^ and 10^−2^ bar (as shown in Fig. 1). Best results were obtained around 830 °C at 10^−3^ bar for a heating rate of 20 °C s^−1^. In the P_O2_-route, the film was heated up to the desired temperature (between 750 °C and 850 °C) at a heating rate between 0.4 °C s^−1^ and 20 °C s^−1^ at an oxygen partial pressure between 2 × 10^−5^ and 10^−6^ bar, and subsequently *P*_O2_ was fast (< 1 s) increased up to the range of 10^−4^–10^−2^ bar. Best results were obtained heating the film up to 835 °C at 20 °C s^−1^ at 10^−5^ bar with a fast change of *P*_O2_ up to 10^−3^ bar. The total pressure was also varied from 10^−2^ to 1 bar depending on the experiment and route used. Films were subsequently oxygenated in a tubular furnace at 450 °C for 200 min in oxygen flowing atmosphere. BaMO_3_ (*M* = Zr, Hf) nanoparticles were prepared following a polyol solvothermal method by mixing Zr or Hf alkoxide precursors and Barium Hydroxide into a mixture of alcoholic solvents^39^. This mixture was treated in an autoclave at 180 °C for 60 min to achieve the crystallization of the nanoparticles. BaZrO_3_ (BZO) of 5 nm of diameter and BaHfO_3_ (BHO) of 4 nm in diameter were obtained in stable colloidal suspensions of 100–120 mM of concentration in ethanol. Afterwards, the colloidal BMO solutions were stabilized in the YBCO precursor solutions to reach the desired final concentration of metal salts with different molar percentages of nanoparticles (up to 32% mol). All solutions were characterized by water content, contact angle, viscosity, metal-stoichiometry and dynamic light scattering (DLS).

The eutectic line of Fig. 1b was determined from data on 3BaO-7CuO binary pyrolyzed powders and thin films on MgO substrates. They were analyzed by thermogramivetry and differential thermal analysis using the Setsys evolution apparatus of Setaram, at subatmospheric conditions *P*_total_ = *P*_O2_ and at a heating rate of 5 °C min^−1^.

### XRD

The phase and texture analysis of the grown films and nanocomposites was analyzed with a high resolution Discover D8 Bruker diffractometer (X-ray energy = 8.049 keV) equipped with a Lynxeye XE energy-dispersive 1-D detector. The quantitative determination of epitaxial fraction of nanoparticles was investigated with a Bruker-AXS D8 Advance diffractometer operating with Cu *K*_α_ radiation equipped with a General Area Detector Diffraction System. Non-uniform r.m.s. strain (nanostrain) was determined from XRD integral-breadth measurements through semiquantitative Williamson–Hall plots using the Discover D8 Bruker system. Debye Scherrer equation was used to determine the nanoparticles size from the (200) reflection.

In situ XRD synchrotron analysis were carried out at DiffAbs beamline at SOLEIL synchrotron with a beam energy of 18 keV, heating ramps of 5 °C s^−1^ and 1 bar of total pressure. An area detector (X-ray hybrid pixel area detector, XPAD) was used^42^ with acquisition times per data point of 500 ms (for phase evolution before YBCO growth) and 100 ms (for YBCO growth), respectively. To follow random phases, grazing XRD scans were recorded, whereas to follow YBCO growth the Bragg conditions with the (005) peak were met (θ–2θ geometry). The experiments were run in a DHS 1100 Anton Paar heater covered with a graphite dome, with maximum heating rates of 5 °C s^−1^ and equipped with a double connection to the vacuum pumps and to the gas inlet system. Electrovalves were used to modulate the total pressure, while an oxygen sensor was used to pre-set the *P*_O2_. For P_O2_-route and T-route, different combinations of N_2_ and Air gases were mixed with a mass flow controller to meet the required conditions. Pole figures (characteristics of the nanoparticles embedded in the YBCO matrix) were recorded during synchrotron experiments at DiffAbs using the XPAD area detector.

### Transmission electron microscopy

In addition, the nanoparticle size in the colloidal solutions were determined by TEM studies with a JEOL of 120 kV 1210 TEM, with a resolution point of 3.2 Å and DLS Zetasizer Nano Zs with measurement range of 0.3 nm–10.0 μm and sensitivity of 0.1 mg mL^−1^. The microstructure, atomic defect structure of films and nanocomposites, and nanoparticles size in the nanocomposite films were studied with a STEM using a FEI Tecnai F20 S/TEM operated in TEM mode at 200 kV, and with a X-FEG gun, a CETCOR probe corrector and a Gatan TRIDIEM 866 ERS energy filter operated in STEM mode at 300 kV. Specimens for STEM were prepared by conventional methods, by grinding, dimpling and Ar ion milling or Focused Ion Beam (FIB). A FIB Zeiss 1560XB Cross Beam was also used to perform and visualize the cross-section of Supplementary Figure 1a.

### Physical properties

Inductive critical current densities were obtained by applying the Bean critical-state model to thin films. The hysteretic magnetization curves were measured with a commercial superconducting quantum interference device (SQUID) magnetometer from Quantum Design equipped with a superconducting magnet of 7 T. The H* parameter was determined from the J_c_(H) curves with a criterion of 0.9 × *J*_c_ at self-field at 5 K. Critical temperature measurements were determined using a PPMS Quantum Design system using the Van der Pauw method and the derivative criterion. The irreversibility line was measured through a bridge of 40 µm in the same PPMS system using a criterion of R(T_irr_)/R(95 K) = 0.001. Film thickness was determined from a three-dimensional profilometer P16 from KLA-Tencor.

## Supplementary information

Supplementary Information

Peer Review File

## Data Availability

The data that support the findings of this study are available from the corresponding authors on reasonable request.
