# Supplementary material for: Ultrafast transient liquid assisted growth of high current density superconducting films
Source: Nat Commun. 2020 Jan 17;11:344. doi: 10.1038/s41467-019-13791-1 (PMC6969047; doi:10.1038/s41467-019-13791-1)
Supplement: Supplementary file 1 — Supplementary Information [file 41467_2019_13791_MOESM1_ESM.pdf]

Supporting information

Ultrafast transient liquid assisted growth of high current density superconducting films

L. Soler[1], J. Jareño[1], J. Banchewski[1], S. Rasi[2,1], N. Chamorro[3,1], R. Guzman[1], R. Yáñez[3], C. Mocuta[4], S. Ricart[1], J. Farjas[2], P. Roura-Grabulosa[2], X. Obradors[1], T. Puig[1 *]

[1] Institut de Ciència de Materials de Barcelona, ICMAB-CSIC, Campus UAB, 08193 Bellaterra, Catalonia, Spain

[2] GRMT, Dept. of Physics, Universitat de Girona, Campus Montilivi, Edif. PII, E17071 Girona, Catalonia, Spain

[3] Departament de Química, Universitat Autònoma de Barcelona, 08193 Bellaterra, Catalonia, Spain

[4] Synchrotron SOLEIL, L'Orme des Merisiers Saint-Aubin BP 48, 91192 Gif-sur-Yvette, France

*corresponding author: teresa.puig@icmab.es

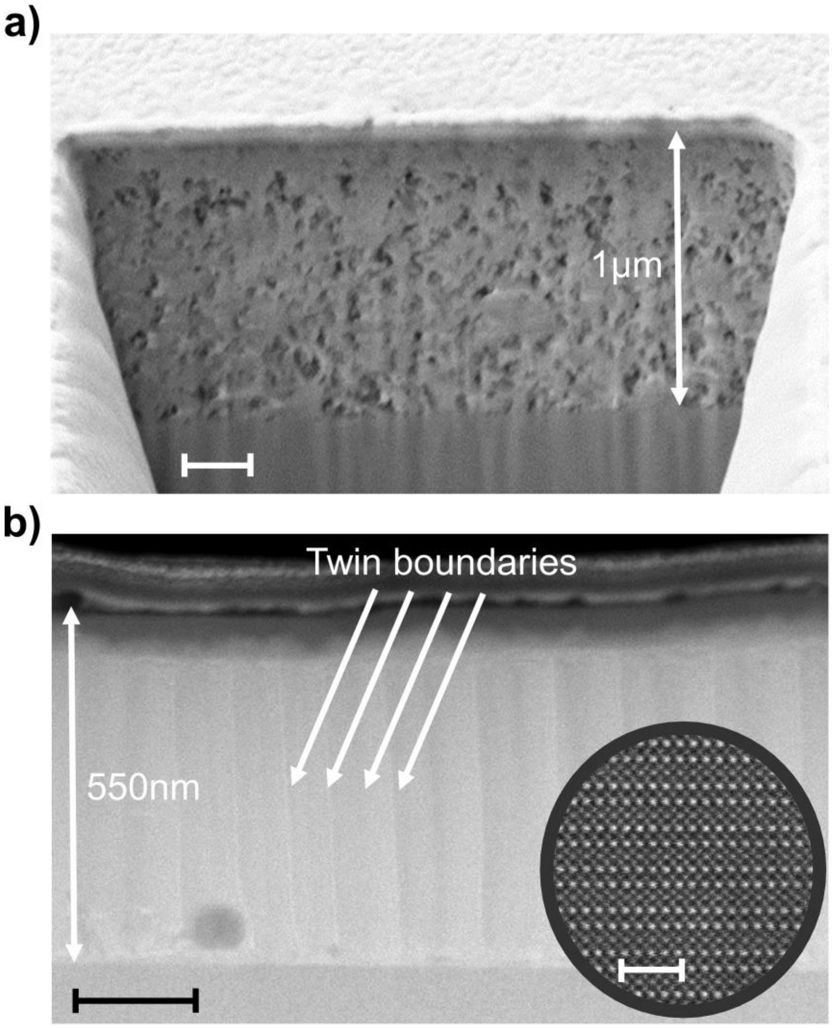

Supplementary Figure 1. Electron Microscopy cross sections of TLAG films. a) Scanning electron microscopy cross section image of a 1 µm thick pyrolyzed ink-jet printed layer. Scale stands for 400 nm b) STEM cross section image of a 550 nm thick YBCO layer with the twin boundaries indicated. The inset is a high resolution STEM image showing the YBCO atomic structure. Scale stands for 200 nm and inset scale for 2 nm.

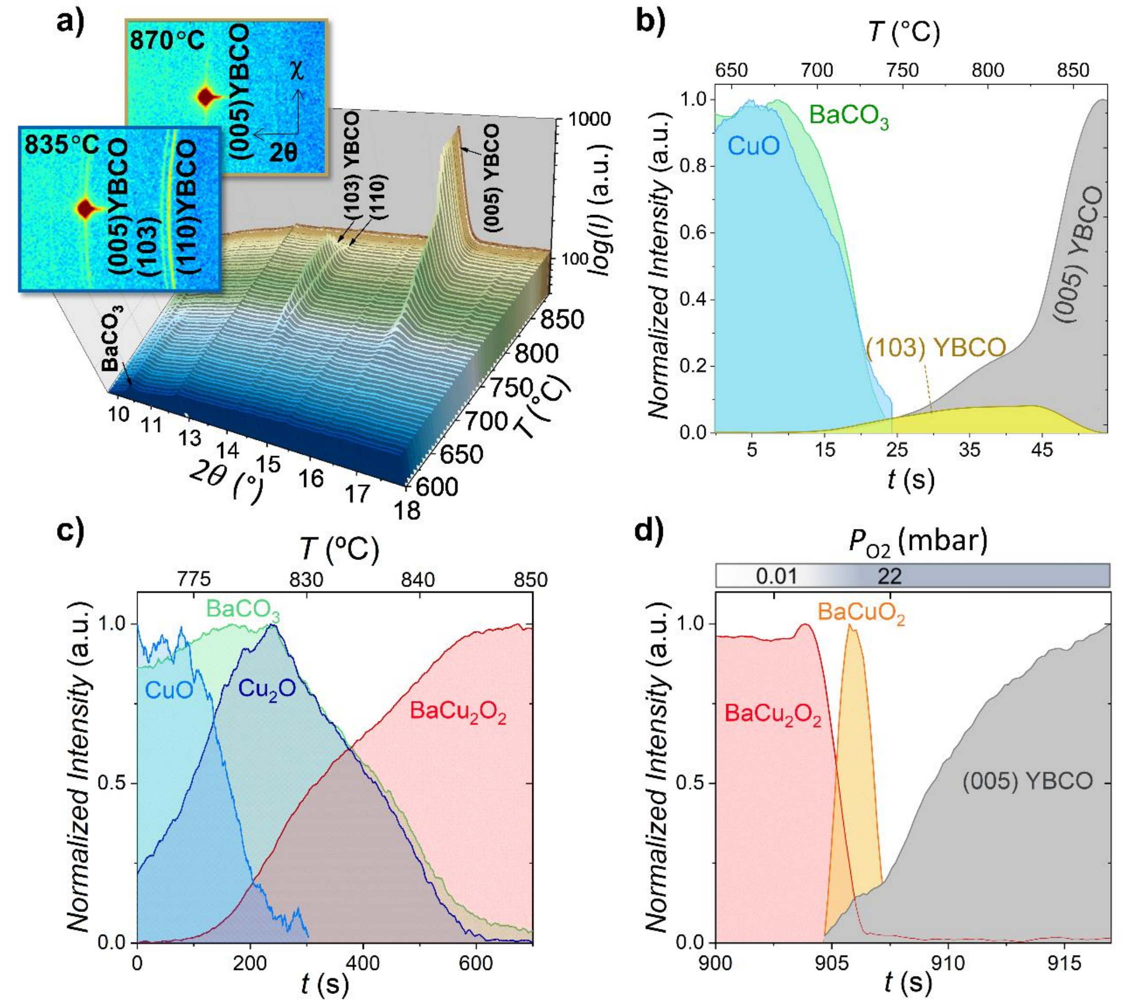

Supplementary Figure 2. In-situ XRD synchrotron analysis of TLAG films. The different reaction mechanisms for YBCO growth described in equations (1) and (2,3) are shown for a x-ray energy = 18 keV. a) XRD intensity versus 2θ and as a function of time for a 2:3 composition (P_{O2}= 10^{-3} bar, 5 ºC s^{-1}), where homogeneous nucleation ((103) YBCO diffraction peak) is seen to disappear at expense of epitaxial growth ((005) YBCO diffraction peak) for a T-route process. Inset shows several selected frames acquired with the 2D detector. b) Evolution of the diffracted intensity originating from different phases for a T-route experiment with 2:3 composition (at P_{O2}= 10^{-3} bar, 5 ºC s^{-1}). c) Similar to panel b) for the initial heating stage of a P_{O2} –route (*dT/dt* = 5 ºC s^{-1} at P_{O2} = 10^{-5} bar) experiment with 3:7 composition. d) Same as panel c) during P_{O2} increase from 10^{-5} bar to $2.2x10^{-2}$ bar (T = 850 ºC). The films were 800 nm thick after pyrolysis, thus giving rise to YBCO grown films of 400 nm in thickness.

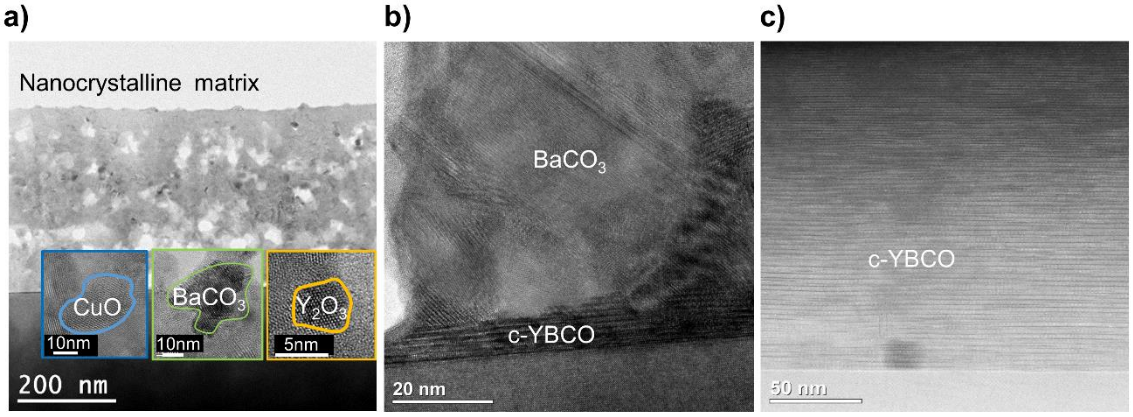

Supplementary Figure 3. STEM cross section analysis for three stages of the TLAG-CSD process for the T-route approach. a) Bright-field transmission electron microscopy (TEM) image after a film pyrolysis at 500 ºC where the porous structure of the different nanocrystalline phases is seen (the insets identify the nanocrystalline phases after the pyrolysis). Shown in grey is the CuO phase, while the $BaCO_3$ matrix is displayed in grey; b) Bright-field transmission electron microscopy (TEM) image after film quenching from 680 ºC (dT/dt=20 ºC s^{-1} and P_{O2}= 10^{-2} bar), where the initial c-axis YBCO nuclei blocked by a $BaCO_3$ grain are observed; c) Scanning transmission electron microscopy (STEM) cross section of the final microstructure of the YBCO film grown at 835 ºC.

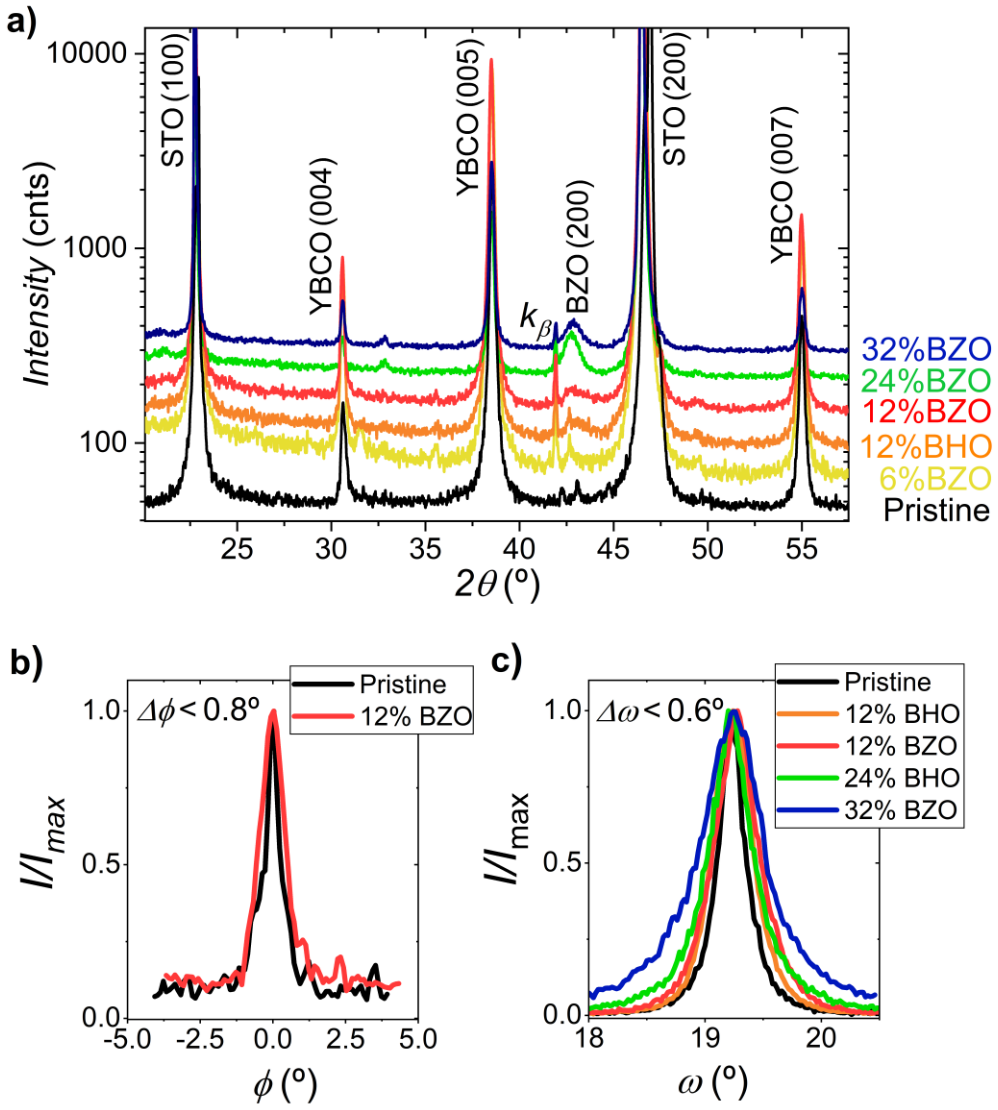

Supplementary Figure 4. X-ray analysis of TLAG nanocomposites. Different nanoparticles content (up to 32 %mol) and composition and a pristine film are evaluated, demonstrating the high quality of the epitaxial growth. a) θ-2θ XRD analysis (X-ray energy = 8.049 keV), where the (00l) peaks of YBCO are identified as well as the (200) reflection of the BZO nanoparticles. b) Azimuthal-scans and c) Rocking curves.

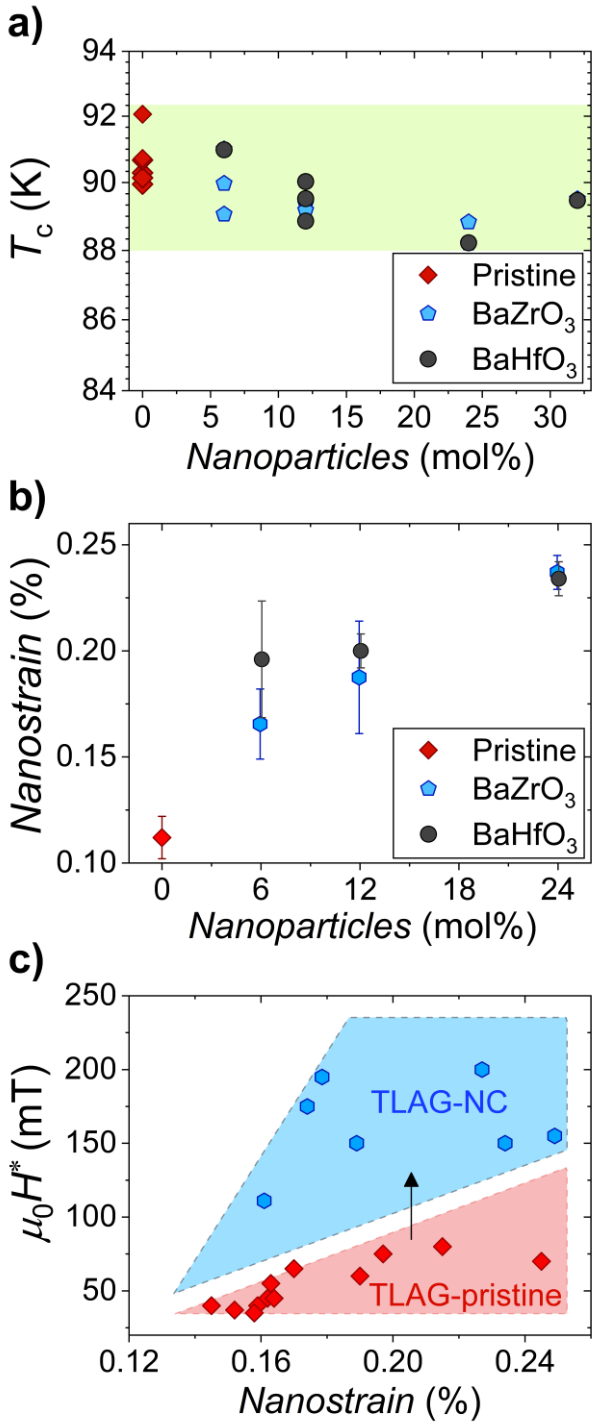

Supplementary Figure 5. Physical properties for TLAG films. Different nanocomposites with nanoparticles of $BaZrO_3$ and $BaHfO_3$, and pristine films are evaluted. a) Superconducting critical temperature, T_c. b) Nanostrain dependence on the concentration of nanoparticles (as indicated). The values were extracted from XRD data, using Williamson-Hall approach. Error bars indicate the results from different samples with the same composition. c) H^* parameter (identifying the crossover field between the single vortex pinning and vortex-vortex interaction regime) as a function of the nanostrain, showing that a pinning mechanism different than nanostrain is active in nanocomposite films.

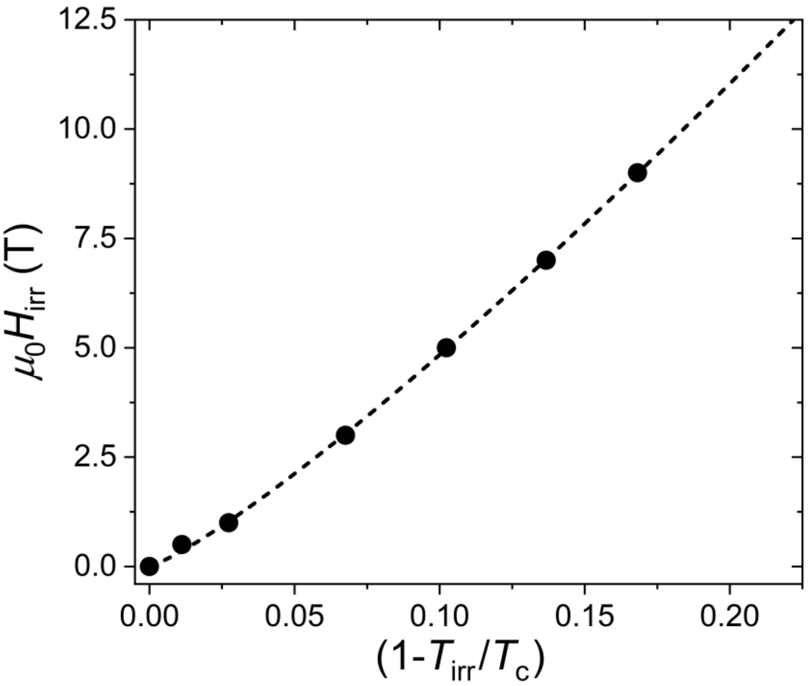

Supplementary Figure 6. Irreversibility line (IL) from the pristine film determined from transport measurements. The criterion used is $R(T_{irr})/R$(95K)=0.001. Filled circles are the measured data and dashed line is the fitting to the equation $\mu_0 H_{irr}$(T)=$\mu_0 H_{irr,0}(1-T_{irr}/T_c)^{\alpha}$, with fitting parameters α=1.19 and $\mu_0 H_{irr,0}$=74.9 T, giving rise to $\mu_0 H_{irr}$(77K)= 8.1 T.
