# Supplementary material for: Ultrafast transient liquid assisted growth of high current density superconducting films
Source: Nat Commun. 2020 Jan 17;11:344. doi: 10.1038/s41467-019-13791-1 (PMC6969047; doi:10.1038/s41467-019-13791-1)
Supplement: Supplementary file 2 — Peer Review File [file 41467_2019_13791_MOESM2_ESM.pdf]

Editorial Note: This manuscript has been previously reviewed at another journal that is not operating a transparent peer review scheme. The manuscript was considered suitable for publication without further review at Nature Communications .
